# Supplementary material for: Overweight, obesity, and thinness among a nationally representative sample of Norwegian adolescents and changes from childhood: Associations with sex, region, and population density
Source: PLoS One. 2021 Aug 3;16(8):e0255699. doi: 10.1371/journal.pone.0255699 (PMC8330951; doi:10.1371/journal.pone.0255699)
Supplement: S1 Fig — (DOCX) [file pone.0255699.s001.docx]

**S1 Fig. Scatterplots^*^ of individual values of height in boys and girls at the 8^th^ grade (age 13 years) assessment.**
^*^The lines are the WHO international growth reference^[[1]](#footnote-2)^ in the traditional 9 centile growth chart format.

1. de Onis M, Onyango AW, Borghi E *et al.* (2007). Development of a WHO growth reference for school-aged children and adolescents. *Bull World Health Organ* 85, 660-667. [↑](#footnote-ref-2)
