# Supplementary material for: Overweight, obesity, and thinness among a nationally representative sample of Norwegian adolescents and changes from childhood: Associations with sex, region, and population density
Source: PLoS One. 2021 Aug 3;16(8):e0255699. doi: 10.1371/journal.pone.0255699 (PMC8330951; doi:10.1371/journal.pone.0255699)
Supplement: S2 Fig — (DOCX) [file pone.0255699.s002.docx]

**S2 Fig.** **Scatterplots^*^ of individual values of BMI in boys and girls at the 8^th^ grade (age 13 year) assessment.**
IOTF, the International Obesity Task Force.

^*^The grey lines are the IOTF growth reference in the traditional 9 centile growth chart format and the red lines are the IOTF cut-off points for thinness grade 1, overweight, obesity, and severe obesity^[[1]](#footnote-2)^.

1. Cole TJ & Lobstein T (2012). Extended international (IOTF) body mass index cut-offs for thinness, overweight and obesity. *Pediatr Obes* 7, 284-294. [↑](#footnote-ref-2)
