# Supplementary material for: Overweight, obesity, and thinness among a nationally representative sample of Norwegian adolescents and changes from childhood: Associations with sex, region, and population density
Source: PLoS One. 2021 Aug 3;16(8):e0255699. doi: 10.1371/journal.pone.0255699 (PMC8330951; doi:10.1371/journal.pone.0255699)
Supplement: S3 Fig — (DOCX) [file pone.0255699.s003.docx]

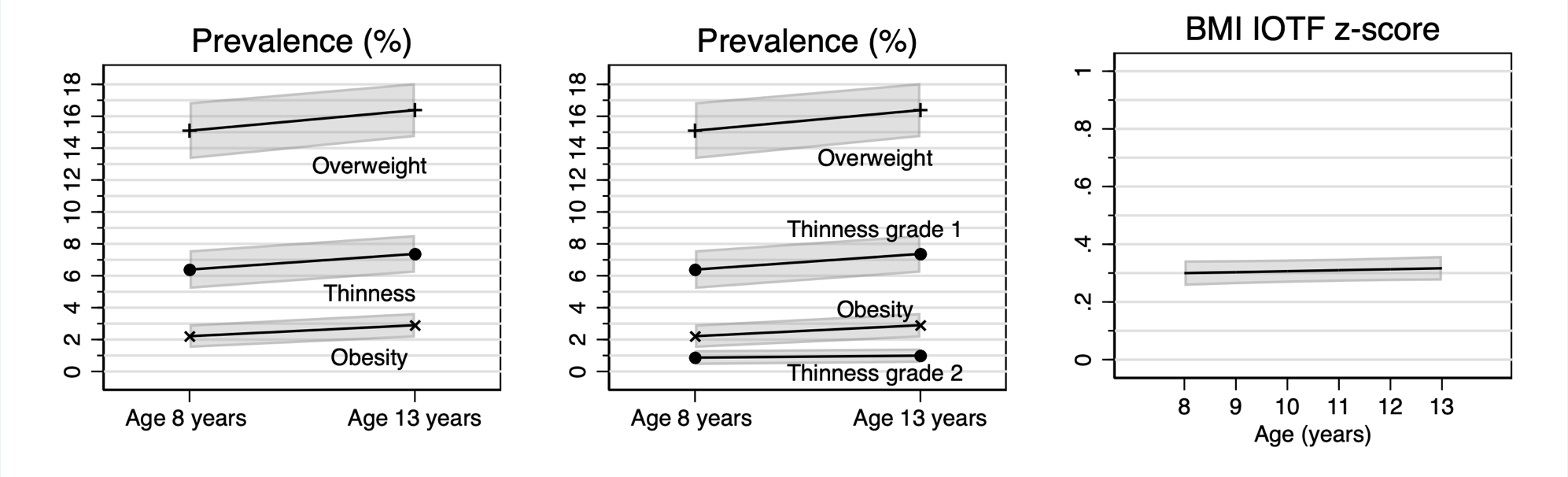


**S3 Fig. Overall predicted prevalence^*^ of IOTF^†^ thinness, overweight, and obesity at 8 years and 13 years (left^†^ and middle**^‡^**), and mean BMI IOTF z-score (right**^**^**).**

BMI, body mass index. IOTF, the International Obesity Task Force.
^*^Presented are the marginal estimates predicted from random effect logistic and continuous multilevel models adjusted for sex (n=1852, 3317 observations).

^†^Categories are inclusive; overweight includes obesity and severe obesity, and similarly for categories of thinness.

^‡^Categories are inclusive for overweight (includes obesity and severe obesity) and obesity; and similarly for categories of thinness grade 2, but exclusive for thinness grade 1.

^**^IOTF z-score: age- and sex-specific standardized z-score calculated from the IOTF LMS parameters^[[1]](#footnote-2)^.

1. Cole TJ & Lobstein T (2012). Extended international (IOTF) body mass index cut-offs for thinness, overweight and obesity. *Pediatr Obes* 7, 284-294. [↑](#footnote-ref-2)
