# Supplementary material for: Overweight, obesity, and thinness among a nationally representative sample of Norwegian adolescents and changes from childhood: Associations with sex, region, and population density
Source: PLoS One. 2021 Aug 3;16(8):e0255699. doi: 10.1371/journal.pone.0255699 (PMC8330951; doi:10.1371/journal.pone.0255699)
Supplement: S4 Fig — (DOCX) [file pone.0255699.s004.docx]

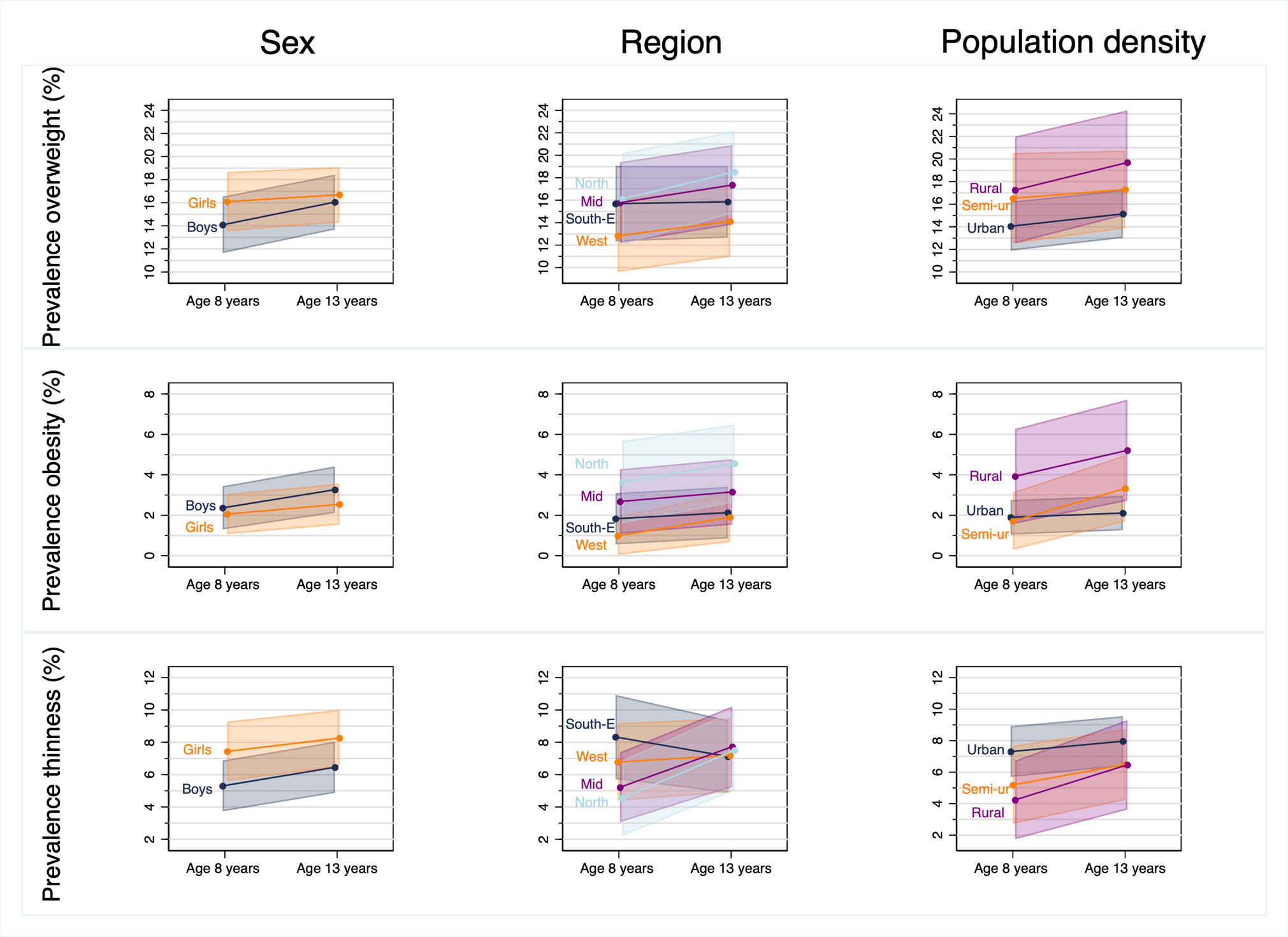


**S4 Fig.** **Predicted prevalence^*^ of IOTF^†^ overweight (top row), obesity (middle), and thinness (bottom) at 8 years and 13 years, by sex (left), region (middle), and population density (right).**

IOTF, the International Obesity Task Force.

^*^Marginal estimates predicted from the random effect logistic regression models in Table S4 (n=1852, 3317 observations), all include an age interaction term. The dots are the point estimates and the shaded area is the 95% confidence interval. The points have been staggered and joined between ages for clarity.
^†^Categories are inclusive; overweight includes obesity and severe obesity, and similarly for categories of thinness.
