# Supplementary material for: Overweight, obesity, and thinness among a nationally representative sample of Norwegian adolescents and changes from childhood: Associations with sex, region, and population density
Source: PLoS One. 2021 Aug 3;16(8):e0255699. doi: 10.1371/journal.pone.0255699 (PMC8330951; doi:10.1371/journal.pone.0255699)
Supplement: S5 Fig — (DOCX) [file pone.0255699.s005.docx]

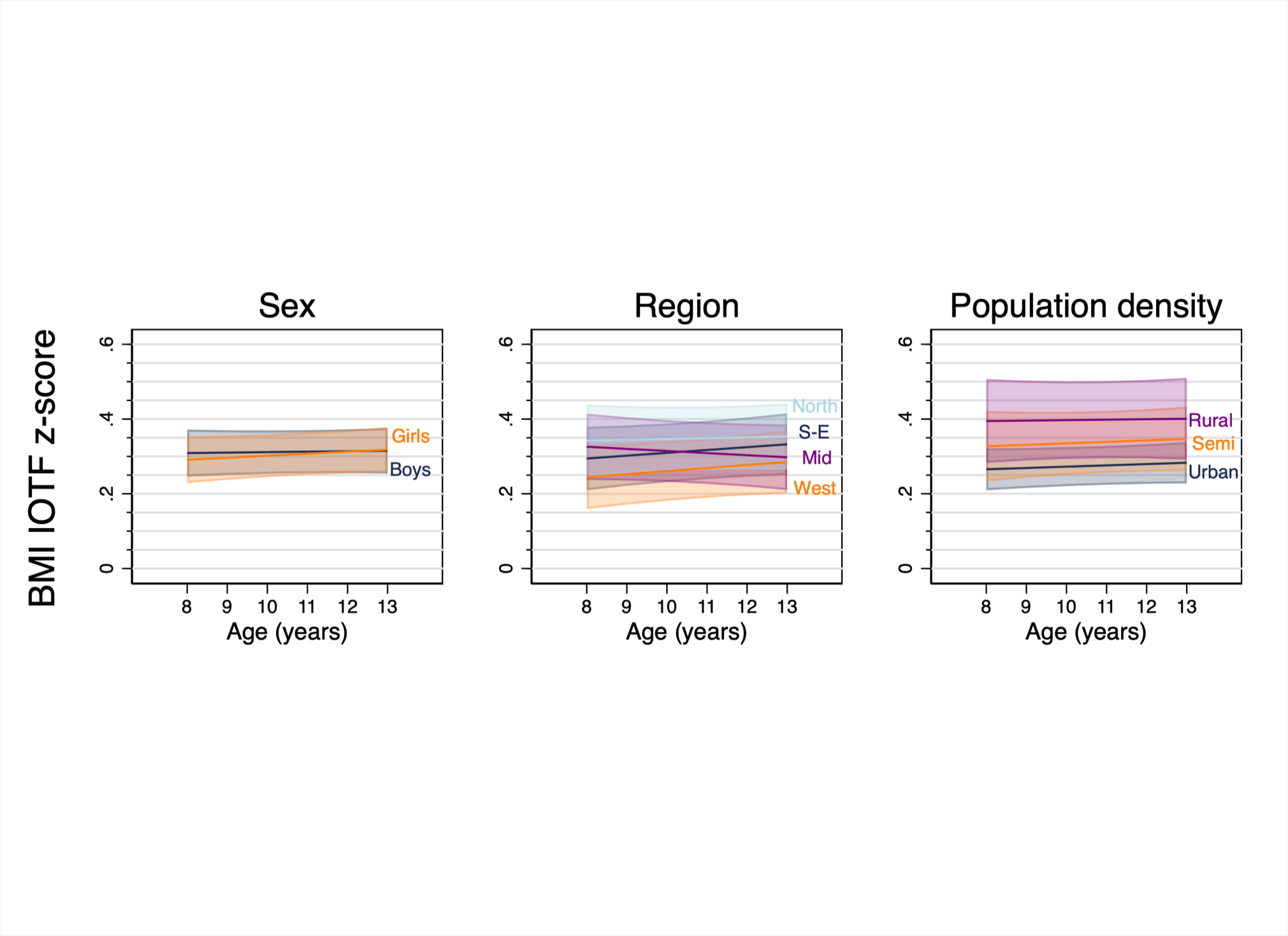


**S5 Fig. Marginal predicted mean BMI IOTF z-score by sex, region, and population density^*^.**

BMI, body mass index. IOTF, the International Obesity Task Force.

^*^Marginal estimates predicted from the random effect regression models in Table S5 (n=1852, 3317 observations), all include an age interaction term. The shaded area is the 95% confidence interval.
