# Supplementary material for: Overweight, obesity, and thinness among a nationally representative sample of Norwegian adolescents and changes from childhood: Associations with sex, region, and population density
Source: PLoS One. 2021 Aug 3;16(8):e0255699. doi: 10.1371/journal.pone.0255699 (PMC8330951; doi:10.1371/journal.pone.0255699)
Supplement: S1 Table — (DOCX) [file pone.0255699.s006.docx]

| **S1 Table. Cross tabulation of region and population density, n (%).** | | | | |  |
| --- | --- | --- | --- | --- | --- |
|  | Urban | Semi-urban | Rural | Total | |
| South-East | 462 (93.3%) | 18 (3.6%) | 15 (3.1%) | 495 (100%) | |
| West | 377 (78.4%) | 36 (7.5%) | 68 (14.1%) | 481(100%) | |
| Mid | 185 (41.9%) | 186 (42.2%) | 70 (15.9%) | 441 (100%) | |
| North | 87 (20.0%) | 216 (49.7%) | 132 (30.4%) | 435 (100%) | |
| Total | 1111 (60.0%) | 456 (24.6%) | 285 (15.4%) | 1852 (100) | |
