# Supplementary material for: Overweight, obesity, and thinness among a nationally representative sample of Norwegian adolescents and changes from childhood: Associations with sex, region, and population density
Source: PLoS One. 2021 Aug 3;16(8):e0255699. doi: 10.1371/journal.pone.0255699 (PMC8330951; doi:10.1371/journal.pone.0255699)
Supplement: S2 Table — (DOCX) [file pone.0255699.s007.docx]

**S2 Table. Median, 2.5^th^ and 97.5^th^ centiles (i.e., 95% reference range) of WHO height z-scores at 13 years (n=1838)^*^.**

|  | Total | | |  | Boys | | |  | Girls | | |
| --- | --- | --- | --- | --- | --- | --- | --- | --- | --- | --- | --- |
|  | 2.5^th^ | 50^th^ | 97.5^th^ |  | 2.5^th^ | 50^th^ | 97.5^th^ |  | 2.5^th^ | 50^th^ | 97.5^th^ |
| WHO height z-score | -1.50 | 0.50 | 2.55 |  | -1.57 | 0.48 | 2.69 |  | -1.24 | 0.52 | 2.29 |
| ^*^Estimates are weighted by the sampling design. We would expect values of -1.96z, 0z and +1.96z for the 2.5^th^ median and 97.5^th^ centiles if the sample perfectly mapped onto the World Health Organization (WHO) reference. | | | | | | | | | | | |
