# Supplementary material for: Overweight, obesity, and thinness among a nationally representative sample of Norwegian adolescents and changes from childhood: Associations with sex, region, and population density
Source: PLoS One. 2021 Aug 3;16(8):e0255699. doi: 10.1371/journal.pone.0255699 (PMC8330951; doi:10.1371/journal.pone.0255699)
Supplement: S3 Table — (DOCX) [file pone.0255699.s008.docx]

| **S3 Table. Prevalence (%, 95% CI)^*^ of exclusive^†^ IOTF overweight, obesity, and thinness at 13 years (n=1838).** | | | | | | | | | | | | |
| --- | --- | --- | --- | --- | --- | --- | --- | --- | --- | --- | --- | --- |
|  | Overweight | | Obesity | | Severe obesity | | Thinness grade 1 | | Thinness grade 2 | | Thinness grade 3 | |
|  | BMI ≥25 to <30 | | BMI ≥30 to <35 | | BMI ≥35 | | BMI <18.5 to ≥17 | | BMI <17 to ≥16 | | BMI <16 | |
|  | % | 95% CI | % | 95% CI | % | 95% CI | % | 95% CI | % | 95% CI | % | 95% CI |
| Overall | 13.3 | 11.2, 15.8 | 2.2 | 1.5, 3.1 | 0.3 | 0.1, 1.0 | 6.1 | 4.8, 7.7 | 1.1 | 0.6, 1.8 | 0.1 | 0.02, 0.9 |
| Sex | | | | | | | | | | | | |
| Boys | 12.5 | 9.8, 15.8 | 2.7 | 1.7, 4.4 | 0.3 | 0.04, 1.7 | 4.7 | 3.1, 6.9 | 0.9 | 0.4, 1.8 | 0.3 | 0.04, 1.8 |
| Girls | 14.1 | 11.2, 17.7 | 1.7 | 0.9, 3.1 | 0.3 | 0.04, 1.9 | 7.5 | 5.5, 10.1 | 1.2 | 0.5, 2.7 | 0.0 | NA |
| Region | | | | | | | | | | | |  |
| South-East | 13.4 | 10.0, 17.8 | 1.8 | 1.0, 3.2 | 0.4 | 0.1, 1.8 | 5.7 | 3.9, 8.4 | 1.2 | 0.6, 2.7 | 0.2 | 0.02, 2.05 |
| West | 12.2 | 9.9, 14.9 | 1.9 | 0.8, 4.5 | 0.0 | NA | 6.3 | 4.3, 8.9 | 0.9 | 0.3, 2.1 | 0.0 | NA |
| Mid | 14.4 | 10.7, 19.2 | 3.0 | 1.6, 5.6 | 0.0 | NA | 6.7 | 4.2, 10.5 | 0.9 | 0.4, 2.2 | 0.2 | 0.03, 1.8 |
| North | 14.0 | 11.4, 17.1 | 4.2 | 2.8, 6.2 | 0.5 | 0.1, 2.3 | 6.9 | 5.0, 9.5 | 0.7 | 0.2, 2.0 | 0.0 | NA |
| Population density | | | | | | | | | | | | |
| Urban | 13.2 | 10.6, 16.2 | 1.7 | 1.0, 2.9 | 0.3 | 0.1, 1.3 | 6.3 | 4.7, 8.3 | 1.2 | 0.6, 2.2 | 0.1 | 0.01, 1.5 |
| Semi-urban | 13.7 | 9.5, 19.4 | 2.9 | 1.5, 5.6 | 0.0 | NA | 5.5 | 3.3, 9.0 | 0.9 | 0.4, 2.2 | 0.0 | NA |
| Rural | 14.0 | 9.9, 19.3 | 5.1 | 3.4, 7.4 | 0.0 | NA | 5.3 | 3.5, 8.0 | 0.5 | 0.1, 3.8 | 0.3 | 0.04, 3.1 |
| BMI: body mass index, kg/m^2^; IOTF, the International Obesity Task Force; NA, Not applicable due to no observations.  ^*^Estimates are weighted by the sampling design.  ^†^Categories are exclusive so overweight does not include obesity or severely obese, similarly for categories of thinness. | | | | | | | | | | | | |
