# Supplementary material for: Overweight, obesity, and thinness among a nationally representative sample of Norwegian adolescents and changes from childhood: Associations with sex, region, and population density
Source: PLoS One. 2021 Aug 3;16(8):e0255699. doi: 10.1371/journal.pone.0255699 (PMC8330951; doi:10.1371/journal.pone.0255699)
Supplement: S4 Table — (DOCX) [file pone.0255699.s009.docx]

| **S4 Table. Associations of sex, region, and population density with IOTF^†^ overweight, obesity, and thinness from 8 to 13 years (n=1852, 3317 observations).** | | | | | | | | | | | |
| --- | --- | --- | --- | --- | --- | --- | --- | --- | --- | --- | --- |
|  | 8 years | | |  | 13 years | | |  | Interaction odds ratio 8 to 13 years^*^ | | |
|  | Odds ratio | 95% CI | P value |  | Odds ratio | 95% CI | P value |  | Odds ratio | 95% CI | P value |
| Overweight, BMI ≥25 | | | | | | | | | | | |
| Sex |  |  |  |  |  |  |  |  |  |  |  |
| Boys | Reference |  |  |  | Reference |  |  |  | Reference |  |  |
| Girls | 1.42 | 0.77, 2.63 | 0.27 |  | 1.11 | 0.64, 1.94 | 0.70 |  | 0.78 | 0.44, 1.41 | 0.41 |
| Region |  |  |  |  |  |  |  |  |  |  |  |
| South-East | Reference |  |  |  | Reference |  |  |  | Reference |  |  |
| West | 0.60 | 0.26, 1.39 |  |  | 0.74 | 0.34, 1.62 |  |  | 1.24 | 0.56, 2.78 | 0.60 |
| Mid | 1.01 | 0.44, 2.62 | 0.50 |  | 1.28 | 0.59, 2.79 | 0.31 |  | 1.26 | 0.57, 2.77 | 0.57 |
| North | 1.07 | 0.44, 2.62 |  |  | 1.53 | 0.71, 3.33 |  |  | 1.43 | 0.61, 3.33 | 0.41 |
| Population density |  |  |  |  |  |  |  |  |  |  |  |
| Urban | Reference |  |  |  | Reference |  |  |  | Reference |  |  |
| Semi-urban | 1.53 | 0.71, 3.30 | 0.33 |  | 1.43 | 0.74, 2.78 | 0.15 |  | 0.94 | 0.45, 1.94 | 0.90 |
| Rural | 1.73 | 0.73, 4.06 |  |  | 2.07 | 0.95, 4.49 |  |  | 1.20 | 0.54, 2.68 | 0.70 |
| Obesity, BMI ≥30 | | | | | | | | | | | |
| Sex |  |  |  |  |  |  |  |  |  |  |  |
| Boys | Reference |  |  |  | Reference |  |  |  | Reference |  |  |
| Girls | 0.78 | 0.24, 2.53 | 0.68 |  | 0.63 | 0.24, 1.68 | 0.36 |  | 0.81 | 0.24, 2.71 | 0.70 |
| Region |  |  |  |  |  |  |  |  |  |  |  |
| South-East | Reference |  |  |  | Reference |  |  |  | Reference |  |  |
| West | 0.36 | 0.05, 2.50 |  |  | 0.82 | 0.18, 3.77 |  |  | 2.27 | 0.31, 16.8 | 0.42 |
| Mid | 1.96 | 0.39, 9.73 | 0.11 |  | 2.02 | 0.49, 8.32 | 0.098 |  | 1.03 | 0.19, 5.54 | 0.97 |
| North | 3.43 | 0.69, 16.9 |  |  | 4.08 | 1.04, 16.0 |  |  | 1.19 | 0.23, 6.22 | 0.8 |
| Population density |  |  |  |  |  |  |  |  |  |  |  |
| Urban | Reference |  |  |  | Reference |  |  |  | Reference |  |  |
| Semi-urban | 0.83 | 0.16, 4.33 | 0.15 |  | 2.31 | 0.70, 7.54 | 0.028 |  | 2.76 | 0.51, 14.8 | 0.24 |
| Rural | 3.82 | 0.91, 16.01 | 0.067 |  | 5.61 | 1.55, 20.3 |  |  | 1.47 | 0.35, 6.12 | 0.6 |
| Thinness, BMI <18.5 | | | | | | | | | | | |
| Sex |  |  |  |  |  |  |  |  |  |  |  |
| Boys | Reference |  |  |  | Reference |  |  |  | Reference |  |  |
| Girls | 1.81 | 0.91, 3.58 | 0.088 |  | 1.56 | 0.87, 2.81 | 0.138 |  | 0.86 | 0.42, 1.77 | 0.70 |
| Region |  |  |  |  |  |  |  |  |  |  |  |
| South-East | Reference |  |  |  | Reference |  |  |  | Reference |  |  |
| West | 0.68 | 0.29, 1.64 |  |  | 1.02 | 0.45, 2.31 |  |  | 1.49 | 0.58, 3.83 | 0.41 |
| Mid | 0.43 | 0.17, 1.09 | 0.15 |  | 1.16 | 0.51, 2.67 | 0.98 |  | 2.70 | 1.00, 7.31 | 0.051 |
| North | 0.34 | 0.12, 0.98 |  |  | 1.10 | 0.48, 2.54 |  |  | 3.22 | 1.06, 9.8 | 0.039 |
| Population density |  |  |  |  |  |  |  |  |  |  |  |
| Urban | Reference |  |  |  | Reference |  |  |  | Reference |  |  |
| Semi-urban | 0.55 | 0.22, 1.36 | 0.13 |  | 0.69 | 0.34, 1.42 | 0.49 |  | 1.25 | 0.48, 3.24 | 0.64 |
| Rural | 0.39 | 0.14, 1.12 |  |  | 0.69 | 0.29, 1.62 |  |  | 1.74 | 0.58, 5.22 | 0.32 |
| BMI: body mass index, kg/m^2^; IOTF, the International Obesity Task Force.  ^*^Estimated from mixed effect logistic models that include an interaction term with age to assess whether the odds have changed from 8 to 13 years.  ^†^Categories are inclusive; overweight includes obesity and severe obesity, and similarly for categories of thinness. | | | | | | | | | | | |
