# Supplementary material for: Overweight, obesity, and thinness among a nationally representative sample of Norwegian adolescents and changes from childhood: Associations with sex, region, and population density
Source: PLoS One. 2021 Aug 3;16(8):e0255699. doi: 10.1371/journal.pone.0255699 (PMC8330951; doi:10.1371/journal.pone.0255699)
Supplement: S5 Table — (DOCX) [file pone.0255699.s010.docx]

| **S5 Table. Associations of sex, region, and population density with BMI IOTF z-score^*^ from 8 to 13 years (n=1852, 3317 observations).** | | | | | | | | | | | |
| --- | --- | --- | --- | --- | --- | --- | --- | --- | --- | --- | --- |
|  | 8 years | | |  | 13 years | | |  | Interaction 8 to 13 years^*^ | | |
|  | Mean difference | 95% CI | P value |  | Mean difference | 95% CI | P value |  | Mean difference | 95% CI | P value |
| Sex | | | | | | | | | | | |
| Boys | Reference |  |  |  | Reference |  |  |  | Reference |  |  |
| Girls | -0.02 | -0.11, 0.07 | 0.69 |  | 0.00 | -0.08, 0.09 | 0.95 |  | 0.02 | -0.04, 0.08 | 0.50 |
| Region | | | | | | | | | | | |
| South-East | Reference |  |  |  | Reference |  |  |  | Reference |  |  |
| West | -0.05 | -0.17, 0.07 | 0.42 |  | -0.05 | -0.17, 0.07 | 0.43 |  | 0.00 | -0.08, 0.09 | 0.96 |
| Mid | 0.03 | -0.09, 0.15 | 0.62 |  | -0.03 | -0.16, 0.09 | 0.57 |  | -0.07 | -0.15, 0.02 | 0.14 |
| North | 0.05 | -0.08, 0.18 | 0.46 |  | 0.02 | -0.10, 0.14 | 0.74 |  | -0.03 | -0.12, 0.07 | 0.57 |
| Population density | | | | | | | | | | | |
| Urban | Reference |  |  |  | Reference |  |  |  | Reference |  |  |
| Semi-urban | 0.06 | -0.05, 0.17 | 0.27 |  | 0.06 | -0.04, 0.17 | 0.22 |  | 0.00 | -0.08, 0.08 | 0.95 |
| Rural | 0.13 | 0.00, 0.25 | 0.043 |  | 0.12 | -0.00, 0.22 | 0.059 |  | -0.01 | -0.10, 0.08 | 0.80 |
| IOTF, the International Obesity Task Force.  ^*^IOTF z-score: age- and sex-specific standardized z-score calculated from the IOTF LMS parameters^[[1]](#footnote-2)^.  ^†^Estimated from random effect multilevel models that include an interaction term with age to assess whether the mean slope from 8 to 13 years differs between categories. | | | | | | | | | | | |

1. Cole TJ & Lobstein T (2012). Extended international (IOTF) body mass index cut-offs for thinness, overweight and obesity. *Pediatr Obes* 7, 284-294. [↑](#footnote-ref-2)
