# Supplementary material for: Overweight, obesity, and thinness among a nationally representative sample of Norwegian adolescents and changes from childhood: Associations with sex, region, and population density
Source: PLoS One. 2021 Aug 3;16(8):e0255699. doi: 10.1371/journal.pone.0255699 (PMC8330951; doi:10.1371/journal.pone.0255699)
Supplement: S1 Text — (DOCX) [file pone.0255699.s011.docx]

**Supporting information**

**S1 Text: Data cleaning**

Data were cleaned using a systematic screening algorithm that made use of logical data checks and the internal consistency of growth for each child (full details are in Wills, 2020^[[1]](#footnote-2)^).

Some errors were able to be cleaned, for example where height was entered in the weight field and vice-versa. In total, twelve errors were unable to be cleaned and were removed at age 8 and 13 years. Of these twelve, two were from the same individual so this child did not contribute to either the cross-sectional or multilevel analyses.

1. Wills AK (2020). Screening & diagnosing errors in longitudinal measures of body size. *medRxiv*, 2020.2011.2019.20234872 [↑](#footnote-ref-2)
