# Supplementary material for: Overweight, obesity, and thinness among a nationally representative sample of Norwegian adolescents and changes from childhood: Associations with sex, region, and population density
Source: PLoS One. 2021 Aug 3;16(8):e0255699. doi: 10.1371/journal.pone.0255699 (PMC8330951; doi:10.1371/journal.pone.0255699)
Supplement: Attachment — Submitted filename: Response to Reviewers.docx [file pone.0255699.s012.docx]

PONE-D-21-08959

*Ethical statement added per journal request:*

*“This study was conducted according to the guidelines laid down in the Declaration of Helsinki and all procedures involving research study participants were approved by the Norwegian Regional Committee for Medical Research Ethics (2017/431). Written informed consent was obtained from all participants and their parents, including consent for the school health nurses to collect previous routine height and weight measurements from school health records. Researchers were provided with de-identified data provided by the Division of Health Data and Digitalisation at the Norwegian Institute of Public Health, which could only be accessed through a secure platform in compliance with the Norwegian privacy regulations.”*

*We have also used PLoS Endnote Style and added details about supporting information at the end of the manuscript.*

***Response to reviewers***

*We have included a few additional references when elaborating on a few discussion points.*


Reviewers' comments and response

**Reviewer #1:** This is a well structured and well written paper describing the results of a study using sound statistic and reporting original findings. I have some minor issues.

*Answer: We would like to thank the reviewer for taking the time to assess our manuscript and giving insightful comments. We have addressed the issues you have raised below.*

Methods
1. Please inform whether only public schools were included in the study or also private school. In any case it should be mentioned whether private schools are relevant in Norway.

*Answer: Both public and private schools could be sampled in the study, however most schools (86% in 2017) in Norway are public. There is little distinction between private and public schools in Norway. We have specified this and that the distinction between public and private school in Norway may not be as important compared to other countries. Page 5, lines 123-124.*

2. It is unclear whether assessment methods of the first (older) measurement of height and weight by School Health Services are comparable to those of the NYGS. Do all participants of the NYGS have 2 measurement points?

*Answer: We thank the reviewer for questioning this important detail and have added specifications of the assessment methods in the manuscript (page 6, lines 140-148). Furthermore, as the measurements from 3^rd^ grade (age 8 years) were collected from the 8^th^ grade participants’ health record, 1478 also had a 3^rd^ grade (8 years) measure. The previous number 1544 was an error, which we now have corrected. We have changed the wording to make this clearer in the manuscript, page 6, lines 144-146.*

*All individuals who had at least one measure (at either age 8 or 13 years) were used in the multilevel models, a total of 1852 individuals (as shown in Fig 1) and specified in Table 1, page 10). From the sample used in the longitudinal analysis, 79% (1467 out of 1852) had measurements at both age 8 and 13. We have included a sentence describing this under sample description, in the result section (page 9, lines 226-228).*

3. Please include information about ethics approval also in the methods section (“This study was conducted according to the guidelines laid down in the Declaration of Helsinki and all procedures involving research study participants were approved by the Norwegian Regional Committee for Medical Research Ethics (2017/431). Written informed consent was obtained from all participants and their parents.”

*Answer: We have inserted this in the method section and added additional information per journal request, page 7, lines 175-182.*

4. Assessment methods should be described more in detail if possible: who measured (how many persons) and with which devices (scale, stadiometer)

*Answer: We have inserted more details based on the assumption that the guidelines were followed. Additional details beyond this are unfortunately unknown. See page 6, lines 140-148.*

5. The definition of urbanity / rurality is somewhat arbitrary and thus hard to compare internationally. Maybe information on population density or other internationally comparable parameters could help

*Answer: The reviewer makes a valid suggestion and we have changed the wording to population density, however low populated areas are described as rural (connected with or like the countryside which have low population density) and urban (connected with a town or city). The relative classification is described under methods, page 7, lines 168-172. Additionally, we have included a sentence pointing out the variety in rural areas between counties in the discussion, page 19, lines 350-352.*

Results
6. The used wording (“increase”, “decrease”) implies that the study found trends. However, two measurement points are not enough to describe trends especially when the assessment methods may not have been identical. Talking about “differences” would be more cautious. This issue could also be included in the “limitations” section of the discussion.

*Answer: We have revised the manuscript to describe the changes in a more cautious ways, as we agree that two measurement points are not enough to describe trends. We also added a sentence under limitations in the discussion section, page 22, lines 427-428.*

Discussion
7. Public Health Implication: The inclusion of additional data (living situation, educational level or migration background of the parents) via record linkage would help to better define potential target populations, e.g., see <https://doi.org/10.4414/smw.2017.14501>

*Answer: The reviewer makes an important suggestion to better define target populations. We have included this in the discussion, page 21, lines 398-401.*

**Reviewer #2:** Reviewer comments on # PONE-D-21-08959 “Overweight, obesity, and thinness among a nationally representative sample of Norwegian adolescents and changes from childhood: associations with sex, region, and urbanicity”

The topic is self is timely relevant and interesting.

*Answer: We would like to thank the reviewer for taking the time to assess our manuscript and giving valuable comments. We have addressed the questions and comments below.*

8. Are children in 8th grade 13 or 14 years old – please check this. In some Scandinavian schooling system preschool is considered as grade 0. To me there is a mismatch.

*Answer: In Norway children usually go directly from kindergarten to school and begin first grade the year the turn 6 years old. There is no grade 0 in Norway, and grade 8^th^ corresponds to the year children turn 13 years old, which is why we the sample consists of 13-year-olds (most 8^th^ graders). As we know this differs between countries, we use age instead of grade when appropriate in the manuscript.*

9. The study design and sampling are well described, assuring population-based sampling. However, when the participation is based on both parent consents this might challenge the population-based sampling as typically the families with higher education will participate. Please add discussion on this to the limitations.

*Answer: We agree with the reviewer that needing consent from both parents might introduce bias and limits the generalizability. However,* we assessed the impact of this by comparing weight measurements at 8 years with a cohort from the NCGS born the same year, which did not need consent from both parents, and the prevalences were similar. However, we were unable to compare cohorts on other factors for example socio-economic status as we lacked this information. *This is addressed under limitations, page 21-22, lines 418-423.*

10. Line 142: Additionally, the school health nurses also reported 3rd grade height and weight measurements (when participants were 7-8 years old -available for 1544 of the 1907 8th graders) recorded by the School Health Service in the adolescent’s health card.--> Exact age of children is needed (decimal age).

*Answer: Due to the school system, children in 3^rd^ grade have already turned 8 years or do it during the fall semester, meaning children could range from 7-8 years as most measurements are conducted during the fall semester. The specified age from the sample included is provided in Table 1 (page 10); a mean age of 8.4 years. We have corrected the wording from “7-8 years old” to “approximately 8 years old” (see page 6, line 144-146), and hope the reviewers finds this, including the information in Table 1, to be satisfying and clear.*

11. Line 157: Similarly, we used the following World Health Organization (WHO) cut-offs for thinness: Thinness grade 1: BMI <18.5 kg/m2; Thinness grade 2: BMI <17 kg/m2; and Thinness grade 3: BMI <16 kg/m2 (18). Good that the authors use thinness instead of underweight. As suggested by Cole (2008) please focus on thinness grade 2 and 3, as it is impractical and confusing to define such a high number of children and adolescents having grade 1 thinness during growth.

*Answer: We acknowledge the reviewers point and have now included thinness grade 2 or grade 3 when looking at differences from 8 years to 13 years in Figure S3 (supporting information) and in the text. However, we could not conduct analysis of grade 2 and 3 stratified across our factors of interest (sex, region, and population density) because the number of individuals were too low to provide any meaningful precision/inference (there were only 14 children (0.93%) classified as thinness grade 2 or 3 at 8 years, and 20 children (1.06%) at 13 years). We still include thinness grade 1 in those parts of the paper that describe differences between sex, region and population density to at least provide some information about this end of the distribution which as we discuss, has been little reported. Few studies present prevalence of thinness and associations, so we consider it a strength to present all grades, especially as this seems to be presented inconsistently in the literature, which make comparisons across studies more difficult. By presenting all grades we present the complete picture. In addition, we corrected errors regarding when thinness is presented as inclusive in the manuscript, and have tried to make it clear, indicated by superscript in tables and figures, when thinness includes all grades (is inclusive) or just present thinness grade 1, 2 or 3. We have also added a comment in the future studies interested in more critical levels of thinness may need substantially larger sample sizes, page 21, lines 401-402.*

Results
12. Misleading sentence: Compared to the WHO growth reference, adolescents were on average 0.5 standard deviation (SD) taller, corresponding to approximately +3.7 cm and +3.5 cm in boys and girls at 13 years respectively (see S2 Table and S1 Fig). These are out of the scope of the paper, please omit.

*Answer: The purpose of providing this information was to describe our sample against a well-used reference population (WHO), we were not interested in making any inference. Hence why the figures illustrating this are in the supporting information. Also, it allows others to see the individual data points. We have combined the original sentence with the earlier paragraph that describes the sample so that it is clearer that this is just describing our sample (page 9-10, lines 240-243). If you still think this should be removed, we would be happy to do so.*

13. Line 234: 7.3% (95%CI: 6.0, 8.8%) had a BMI below the grade 1 thinness threshold and 0.1% (95% CI: 0.02, 0.9%) had grade 3 thinness (Table 2). On purpose of not, thinness grade 2 is left out. Why? Now, thinnes is written with small capital – be consistent please.

*Answer: This was not done purposefully, and we have included the prevalence of thinness grade 2 in the text (page 11, lines 251-252). We have also fixed the consistency regarding capital letters throughout the manuscript.*

14. The S3 Figure presenting prevalence of overweight, thinness and obese is misleading, as obese are in a way reported twice. BMI z-score on the other side of the panel is ok.

*Answer: We have clarified that these categories are inclusive in all tables and figures so that there is no ambiguity. We think that presenting inclusive IOTF categories (≥25; ≥30 etc.) makes more numerical and substantive sense. The interest was in the difference (change) from childhood to adolescence in % children falling above overweight (BMI ≥25), or falling above obese (BMI ≥30), i.e. it captures the changing mass of the distribution above well-defined and accepted cut-points – this is lost if we restrict to overweight only. Further, almost all papers report inclusive categories and the paper outlining the method (Cole et al. 2012^[1]^) also uses the inclusive categories as an exemplar reporting template.*

15. Line 257: Only the relationship between region and IOTF thinness grade 1 showed any evidence of having a different association between age 8 and 13 years (age interaction)…–
As mentioned earlier, I suggest to combine 2 and 3 grades and leave grade 1 out.

*Answer: As we have noted above, we acknowledge the reviewer’s important point, and have now conducted this analysis to look at difference from 8 to 13 years for thinness grade 2 or 3, included under results, page 14, lines 265-267. For the rest of the analysis of thinness by sex, region and urbanicity/population density we examine grade 1 thinness due to the small number in our sample with grade 2 or 3 thinness – see comment above. Therefore, we had to combine thinness in to one whole category in parts of the paper. This is indicated by superscripts in tables and figures. Still, we would like to thank the reviewer for pointing this out. As there were errors and possible misconceptions in the presentation of thinness, we have corrected this in the text.*

Discussion
16. The authors point, that prevalence of ov/ob remains at the same level during the follow-up – based on cross-sectional observations at two time points.
With follow-up data of same individuals, you may show how many are consistently ov/ob during the follow-up – which is more informative than this. Alternatively, how many becomes ov/ob or the opposite will support your story of stable weight status. I would direct the discussion to this direction than on plain prevalence.

*Answer: We think this is a misunderstanding of the design of our study which is hopefully clarified with our comments above. The data are in fact longitudinal – the majority of individuals (almost 80%) included in our analysis investigating differences from 8 to 13 years had observations at both timepoints. The strength of the multilevel models that we use in this paper is that they include every individual as long as they have at least one measure of the outcome. So, a further 300 or so (approximately 20%) with only one measure, still contributed to our estimates of the change from 8 to 13 years and our estimates of the relationship between sex, region and population density from 8 to 13 years. This sort of modelling minimizes bias due to selection on complete cases and incorporates the individual’s with fewer (one) observation in a statistically principled missing at random way, which is a less severe assumption compared to an analysis that would only include the complete cases. We appreciate the reviewer’s insightful suggestion and agree that further exploration of consistency of overweight and obesity among individuals are valuable. Nonetheless, the suggestion to examine tracking within an individual is a very important one, we think however, that this merits exploration in another paper as the aims are substantively different and outside the scope of our objective. For this reason, we chose not to conduct these analyses, but have emphasized this under discussion, page 21, lines 408-411.*

17. Line 307..the increases were small and statistical evidence was lacking… omit the sentence. Only significant changes worth mentioning.

*Answer: We have removed the sentence.*

18. Line 325: We were unable to detect differences in ov/ob prevalence between boys and girls, which previously have been reported among Norwegian 8-, 10-12-, and 15-year-olds, with more girls than boys having ov/ob (4, 21, 22). Any explanation for this? We see sex-differences when using WHO reference system instead of IOTF, please discuss.

*Answer: As we present ov/ob prevalences according to IOTF reference, we consider it outside the scope of this paper to discuss differences between the WHO and IOTF reference system. We have chosen to compare our findings with studies using the same reference system, to make it easily comparable. Nonetheless, we agree with the reviewer that our findings should be commented upon, and we have included this in the revised manuscript. Page 18-19, lines 343-345.*

19. Line 327: Also, some speculation on contributing factors for higher prevalence of ov/ob in rural and North Norway. Are socioecomical backgrounds or higher education level of families different in these areas compared with urban and West-Norway? Some of these speculations are presented in line 370, reorganize the Discussion and add references, please.

*Answer: We have elaborated on possible explanations of our findings in the discussion; page 19, lines 349-357.*

20. Line 359: The authors wrote: “Although previous studies may have indicated a possible stabilization in secular trends of ov/ob, the prevalence of ov/ob in Norway is already high at age 8 years compared to prevalence in previous decades (1993-2000), and it remains high in adolescence as shown here (31). Thus, policymakers should strengthen the prevention and the health service should increase early treatment of ov/ob among children and adolescents.”

Consider the place of the citation.
I think the authors might draw too early conclusions on this. As the children are growing at 13 years of age, especially boys have not at age 13 undergone puberty. Growth might normalise the weight. Further evidence on stable weight status is needed.

*Answer: We thank the reviewer for this detail and have moved the reference and specified that the prevalence seems to be remaining at this level in adolescence as shown in the current study (page 20, lines 387-390). Additionally, we have included a sentence emphasizing the limitation of using two measurement points when describing age-related patterns under limitations, page 22, lines 427-428.*

21. Line 388: First, we needed consent from both parents, which meant we were unable to collect data from some of the 13-year-olds. Quite strict, in Finland consent from one parent is enough!

*Answer: We agree that this is strict, and this has not been necessary in other, previous studies measuring children. However, this was non-debatable requirement by the Norwegian Regional Ethical Committee for Medical Research.*

1. Cole TJ, Lobstein T. Extended international (IOTF) body mass index cut-offs for thinness, overweight and obesity. Pediatr Obes. 2012;7(4):284-94.
